# Early Onset of Liver Steatosis in a Japanese Girl with Maturity-Onset Diabetes of the Young Type 3 (MODY3)

**DOI:** 10.4274/Jcrpe.584

**Published:** 2012-06-09

**Authors:** Akie Nakamura, Katsura İshidu, Toshihiro Tajima

**Affiliations:** 1 Hokkaido University School of Medicine, Department of Pediatrics, Sapporo, Japan; +81 11 706 5954+81 11 706 7898ajeari@med.hokudai.ac.jp

**Keywords:** Gene mutation, liver disease, MODY3, steatosis

## Abstract

Maturity-onset diabetes of the young type 3 (MODY3) is caused by heterozygous mutation in the HNF1A gene. Liver adenomatosis has been reported in MODY3 patients. The patient reported in this paper is a Japanese girl who first developed hepatomegaly, fatty liver, and hepatic dysfunction at age 5 years. Liver biopsy demonstrated steatosis and degeneration of hepatocytes. At that time, blood glucose and HbA1c levels were within normal ranges. Elevated HbA1c was noticed 4 years later, but islet cell and glutamic acid decarboxylase antibodies were not detected in the serum. Therefore, MODY3 was suspected and subsequent analysis of the HNF1A gene identified a heterozygous germline splice donor-site mutation in intron 9. MODY3 patients should be screened by non-invasive liver imaging, and careful follow-up of liver disease should be performed.

**Conflict of interest:**None declared.

## INTRODUCTION

Hepatocyte nuclear factor-1α (HNF-1α) is a homeodomain transcription factor expressed in a variety of tissues (including liver, pancreas and gut). It regulates a large number of liver-specific genes as well as pancreatic genes involved in glucose metabolism and transport (1,2). In humans, heterozygous germline mutations of the HNF1A gene, encoding HNF-1α, are the cause of maturity-onset diabetes of the young type 3 (MODY3) (2,3). MODY3 is characterized by an onset usually before the age of 25 years, dominant inheritance, and a progressive β-cell failure. However, phenotypic variability is also reported in MODY3 (2,4,5,6). Some patients with MODY3 are controlled with diet and physical exercise, while some require insulin. Furthermore, proliferative retinopathy has been observed frequently in patients with MODY3 (6).

It has been also reported that somatic biallelic inactivation mutations of HNF1A cause hepatocellular adenomas and liver adenomatosis, suggesting that this gene acts as a tumor suppressor in the liver (7,8). Furthermore, cosegregation of liver adenomatosis and diabetes in 4 families and 1 patient harboring germline HNF1A mutations has been described (9,10,11). However, to our knowledge, there have been no reports of liver steatosis in young children who have the HNF1A mutation.

Herein, we report the case of a Japanese girl in whom liver dysfunction and steatosis occurred in early childhood. She later developed diabetes, and germline mutation of the HNF1A gene was subsequently identified.

## CASE REPORT

**Figure 1:**
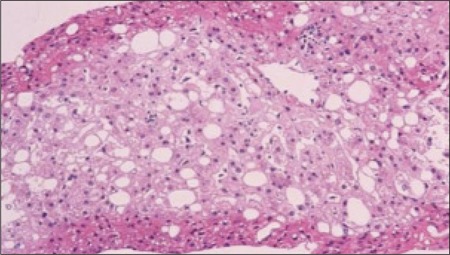
Hematoxyline-eosin staining of the liver biopsy specimen (x20)showing macrovesicular steatosis

The patient is now 20 years old; she is the second child of healthy non-consanguineous parents. There was no family history of liver disease, diabetes, or hypercholesterolemia. Since birth, her general health had been good; however, hepatomegaly was noticed during a routine health care visit and therefore, at 5 years of age, the patient was referred to our hospital. At that time, her height was 100.6 cm (-2.0 SD as compared to a normal Japanese girl) and her weight was 14.1 kg (-1.8 SD as compared to a normal Japanese girl) (BMI= 14.1 kg/m2). A physical examination revealed hepatomegaly with moderate elevation of serum transaminases. Jaundice and splenomegaly were absent. She was admitted for further evaluation . Laboratory findings revealed liver dysfunction and hypercholesterolemia (AST 211 IU/L, ALT 321 IU/L, lactate dehydrogenase 914 IU/L , alkaline phosphatase 971 IU/L, leucine aminopeptidase 272 IU/L, ?-glutamyl transpeptidase 10 IU/L, total cholesterol 207 mg/dL, HDL-cholesterol 45 mg/dL, triglyceride 142 mg/dL, and total bile acid 5.5 mg/L). Serum anti-nuclear antibody, anti-smooth muscle antibody, and anti-DNA antibody were negative. Her fasting blood glucose was 91 mg/dL and within the normal range. Glucosuria was detected; however, an oral glucose tolerance test (OGTT) showed normal glucose levels and insulin responses (Table 1). Abdominal computed tomography (CT) revealed hepatomegaly with diffuse low density and no mass, suggesting fatty liver change. Ultrasonography and CT demonstrated no abnormalities in other organs. It was suspected that the patient had some degree of liver disease, and a liver biopsy was performed. Biopsy specimens showed vacuolar degeneration of individual hepatocytes and macrovesicular steatosis (Figure 1). Hepatitis, drug-induced disease, and congenital metabolic diseases were excluded, and the origin of the liver disease remained unknown. As the cause of liver disease was not determined, we treated the patient for hypercholesterolemia with ethyl icosapentate, which is not hepatotoxic (12). Post-treatment, her serum cholesterol level had decreased to almost within the normal range. Regarding hepatic dysfunction, the patient’s serum transaminase levels were reduced but continued to fluctuate at the upper end of the normal range. A CT scan showed improvement of fatty change in the liver. When the patients was 9 years of age, it was noticed during a routine laboratory evaluation that she had elevated HbA1c (7.3%) and she was reevaluated for diabetes. At this time, her height was 127.4 cm and her body weight was 25.7 kg (these measurements correspond to -1.0 SD for a normal Japanese girl). BMI was calculated as 15.9 kg/m2. The second OGTT showed reductions in both glucose tolerance and insulinogenic index (Table 1). The peak level of serum C-peptide after glucagon stimulation remained normal (3.9 ng/mL, normal range >2.0 ng/mL). Islet cell antibodies (ICAs) and glutamic acid decarboxylase (GAD) antibody were not detected in the serum. At this time, she was suspected to have MODY3 because of liver disease and negative auto-antibodies. It has been reported that sulphonylureas are effective in the treatment of patients with MODY3 (13); however, the use of sulphonylureas in children is not approved by the health insurance authorities in Japan. Since the patient had a reduced insulinogenic index, insulin treatment in a total daily dose of 9 units prior to each meal was initiated. Since that time, the patient’s diabetes has been under good control and her liver dysfunction has been normalized. She is now 20 years old, and her total daily insulin requirement has increased to 36 units, while her HbA1c levels range from 6.5% to 7%. We could not obtain patient and parental consent for a second biopsy.

Because the clinical course led to a probable diagnosis of MODY, we analyzed HNF1A, 4A and -1B by polymerase chain reaction and direct sequencing, according to a previous report (14). Sequence analysis of HNF1A identified a heterozygous mutation at the consensus splice donor site of intron 9, which has been previously reported in a MODY3 family (2). Neither of the patient’s parents had this base change, indicating that the mutation occurred de novo. However, we could not perform an analysis of somatic mutations of HNF1A in liver tissue, which was previously biopsied, because we did not have access to the samples.

## DISCUSSION

Herein, we report the case of a Japanese girl who showed hepatosteatosis in early childhood before the onset of diabetes caused by HNF1A mutation. Her liver biopsy demonstrated vacuolar degeneration of individual hepatocytes and macrovesicular steatosis. Reznik et al (10) described the pathology of liver adenomatosis caused by the HNF1A mutation, noting that the hepatocytes were both swollen and vacuolated with an excessive cytoplasmic and nuclear lipoid accumulation and glycogen accumulation. Bacq et al (9) reported familial liver adenomatosis and MODY3 caused by HNF1A mutation. The pathological findings were macro- and microvesicular steatosis and clear hepatocytes. Thus, the microscopic findings in these 2 studies are very similar to our findings. Repeated imaging of the liver in our patient did not show any adenomatous regions, and therefore, a diagnosis of liver adenomatosis was not made. Although we could not determine the somatic mutation in the liver, the early development of liver disease may be due to a second somatic mutation of HNF1A in liver tissue. Therefore, careful follow-up of liver imaging has been continued.

The coexistence of diabetes and liver adenomatosis caused by HNF1A mutation in 4 families and in 1 sporadic patient has been reported (9,10,11). As liver adenomatosis sometimes causes a fatal outcome, screening by liver imaging is warranted for MODY3 patients.The age at which patients were diagnosed as having liver adenomatosis was variable (9,10,11). The youngest patient with liver adenomatosis described to date was 14 years old. This female patient was first found to have liver adenomatosis and was diagnosed with diabetes one year later. In another family, one patient developed liver adenomatosis at the age of 17 years and the diagnosis of diabetes was established when the patient was 32 years old (10). Thus, some patients harboring the HNF1A mutation develop liver disease before the onset of diabetes. These findings reinforce the importance of screening for diabetes and germline HNF1A mutation in patients and families with liver adenomatosis.

In conclusion, MODY3 patients should be screened using liver function tests and non-invasive liver imaging, and careful follow-up should be performed. Conversely, if patients with fatty change of the liver of unknown origin are seen in childhood, HNF1A defects should be considered to be one of several causes of fatty liver.

## Figures and Tables

**Table 1:**
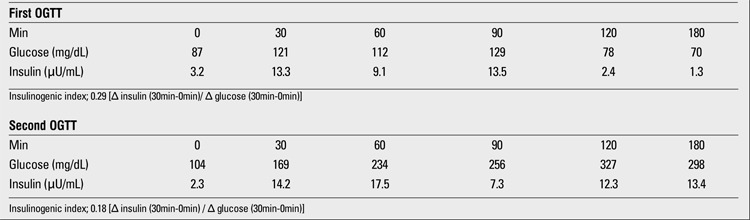
Results of oral glucose tolerance tests (OGTT)
